# Early extubation is associated with improved outcomes after complete surgical repair of pulmonary atresia with ventricular septal defect and hypoplastic pulmonary arteries in pediatric patients

**DOI:** 10.1186/s13019-021-01416-y

**Published:** 2021-03-19

**Authors:** Yinan Li, Yuan Jia, Hongbai Wang, Xie Wu, Shoujun Li, Fuxia Yan, Su Yuan

**Affiliations:** 1 Department of Anesthesiology, Fuwai Hospital, National Center of Cardiovascular Diseases, Chinese Academy of Medical Sciences and Peking Union Medical College, 167 Beilishi Road, Xicheng District, Beijing, 100037 China; 2 Center for Pediatric Cardiac Surgery, Fuwai Hospital, National Center of Cardiovascular Diseases, Chinese Academy of Medical Sciences and Peking Union Medical College, Beijing, 100037 China

**Keywords:** Pulmonary atresia, Ventricular septal defect, Early extubation, Multistage rehabilitation, Medical cost

## Abstract

**Background:**

The aim of this study was to investigate the impact of an early extubation strategy on outcomes following complete repair of pulmonary atresia, ventricular septal defect, and hypoplastic pulmonary artery.

**Methods:**

One hundred thirteen patients undergoing complete repair surgery of pulmonary atresia, ventricular septal defect, and hypoplastic pulmonary artery between 2016 and 2018 were included in our retrospective propensity-score matched study. Propensity score matching was conducted in 1 to 2 ratio to balance the covariables impacting on clinical outcomes between groups. The primary outcomes were defined as length of intensive care unit stay, postoperative length of hospital stay and in-hospital medical cost. The secondary outcomes included postoperative complications such as re-intubation, re-exploration, in-hospital mortality, arrhythmia and etc.. In addition, blood product consumption were also abstracted.

**Results:**

Compared with matched controls, patients in the early extubation group were demonstrated with a significant reduced length of intensive care unit stay (Median: 1.9 d νs. 4.1 d, *p* = 0.039), postoperative length of hospital stay (Median: 9.0 d νs. 17.0 d, *p* = 0.007) and in-hospital medical cost (Median: 69.5 × 1000CNY νs. 113.6× 1000CNY, *p* = 0.041). As for the postoperative complications, the occurrence of re-intubation, re-exploration, in-hospital mortality, arrhythmia and renal replacement therapy was similar between groups. However, pulmonary complications (*p* = 0.049) were with a significantly lower rate in the early extubation group. In addition, fresh frozen plasma (*p* = 0.041) transfusion volume were significantly reduced in the early extubation group rather than packed red blood cells and platelets.

**Conclusions:**

Early extubation following complete repair of pulmonary atresia improved clinical outcomes and reduced in-hospital medical cost without increasing any postoperative complications.

## Background

Extubation is a critical event for the pediatric patients during the postoperative period of congenital heart surgery with cardiopulmonary bypass (CPB). Immediate extubation in the operating room following pediatric cardiothoracic surgery was firstly reported by Barash et al. in 1980 [1]. Despite of its development during the past decades, the risk-benefit profile was still uncertain in the children with complex abnormalities especially those with abnormal pulmonary blood flow or ventricular dysfunction [2–5]. Pulmonary atresia (PA) with ventricular septal defect (VSD) is a rare form of congenital heart disease with an incidence of 0.7 per 10,000 live births, 20 to 40% of which is complicated with hypoplastic or even absent pulmonary arteries (PAs). Major aorto-pulmonary collaterals (MAPCAs) provide the partial or entire source of PAs. The preferred surgical management in our institution is multistage pulmonary artery rehabilitation [6]. Due to long CPB, high incidence of 22q11 microdeletion related immunology system deficiency and surgery related pulmonary contusion or hemorrhage, postoperative ventilation was historically considered as an important component of patient stabilization [7]. However, as is known to all, mechanical ventilation may decrease the system venous return and cardiac output, incline to more positive fluid therapy and vasoactive agent. Excessive fluid and inproper vasoactive agent are both independent risk factors to predict the re-intervention of MAPCAs and prolonged length of intensive care unit stay (LOIS) in patients with PA/VSD. As a relatively large center with over 40 repairs yearly, we would like to assess in our study whether early extubation is associated with better outcomes and lower medical expense of patients with PA/VSD.

## Methods

### Study and patients

In this retrospective study, consecutive patients beyond neonatal stage (>4 weeks) with PA/VSD and hypoplastic PAs between January 2016 and December 2018 were included. The institutional review board and the institutional ethics committee approved the study. The need for individual consent was waived for this observational study. All these patients underwent the multistage rehabilitation and finally received complete repair. Patients without confluent PAs or with malformation of coronary arteries were excluded from this study.

### Surgical technique and anesthesia management

The multistage treatment for PA/VSD to achieve a complete repair consisted of right ventricle to pulmonary artery (RV-PA) connection, MAPCAs occlusion/ligation and PAs angiography to promote a reasonable pulmonary vascularization. And then a complete repair was performed with the closure of VSD, establishment of RV-PA continuity and elimination of extracardiac sources of pulmonary arterial blood flow. There were no major changes in surgical techniques over the study period. The approaches of our institution to extubate were varied from anesthetists. Some anesthetists were positive to carry out on-table extubation or extubation within 6 h after surgery, while others were inclined to transfer the patients to the ICU and leave the timing of extubation determined by attending physicians based on the clinical course. Early extubation has been defined in previous report (within the operating room, ≤6 h post-operatively, or ≤ 24 h post-operatively). Our institution was inclined to include the patients extubated within the operating room or within 6 h postoperatively as early extubation subjects. Whether the early extubation strategy would be implemented were determined by the anesthetist and the surgeon together. Anesthetic management was adapted accordingly. For the whole anesthetic procedure, in addition to midazolam, cis-atracurium, inhaled sevoflurane and fentanyl, dexmedetomidine was either used in every patients. In the early extubation team, the total dose of fentanyl and midazolam were limited to 15 μg/kg and 0.3 mg/kg/h respectively. Postoperatively, patient controlled intravenous analgesia device loaded with sulfentanyl, antiemetics and dexmedetomidine were either used to maintain sufficient analgesia and allow for spontaneous breathing. Propofol was continuous infused before successful extubation. Bolus morphine or non-steroidal analgesics were both the rescue therapy for preference to cure postoperative acute pain. Three times per day’s evaluation of whether the patient was ready to be extubated, which consisted of sinus rhythm, low inotropic support, normothermia and no early complications, were made by the surgeons and attending physicians of ICU. Moreover, if all aspects of assessment, spontaneous breathing and stable hemodynamics were satisfied, the early extubation were implemented in the ICU. The satisfied spontaneous breathing included that respiratory frequency greater than 16 breaths/min, tidal volume > 6 ml/kg and PaCO2 < 45 mmHg.

### Variables and outcomes

For the analysis, the patients cohort was divided into an “early extubation” group defined as patients extubated either on-table or ≤ 6 h postoperatively, and a “standard extubation” group, defined as having mechanical ventilation for more than 6 h postoperatively.

The patients demographics including age, gender and weight were collected. Preoperative variables including American Society of Anesthesiologists (ASA) Grade, diagnosis, McGoon index, previous sternotomy and angiographic results were extracted from the medical record either. Intraoperative variables including cardiopulmonary bypass (CPB) time, aorta cross-clamping (ACC) time, the lowest temperature, the implemented surgery procedure and RV/LV pressure ratio. In addition, variables regarding the postoperative course were extracted and analyzed.

Our primary endpoints were medical expense, LOIS and postoperative length of hospital stay (pLOHS). Secondary endpoints were postoperative complications, including in-hospital mortality, re-intubation (defined as unplanned re-intubation with 24 h after extubation), re-exploration (defined as sternotomy and re-exploration for bleeding within 72 h postoperatively), lung complications (consisting of pneumonia, atelectasis and a large amount of secretions requiring frequent nasotracheal suction), arrhythmia (including junctional ectopic tachycardia and temporary atrioventricular conduction block) and renal replacement therapy (RRT), and postoperative blood products consumption (packed red blood cells (PRBCs), fresh frozen plasma (FFP) and platelets volume standardized by patients’ weight). Re-intubation in 24 h after extubation were regarded as the most important variable to determine the safety of early extubation protocol in our institution.

### Propensity score matching

We used propensity score-matched analyses to match each patient implemented with early extubation with 2 controls. Based on existing knowledges, we involved the following factors demonstrated related to the prolonged LOIS post-pediatric cardiac surgery in the propensity score, including age, weight, gender, major aorta-pulmonary collaterals (MAPCAs), previous sternotomy, intraoperative vasoactive-inotropic score (VIS), CPB time, ACC time, RV/LV pressure ratio, delayed sternal closure, McGoon index and fluid balance within 72 h postoperatively. The patients were matched using a greedy distance matching algorithm. Successful matches were those with estimated propensity score logits within 0.2 standard deviations of the logit of the propensity score. We calculated the absolute standardized mean difference (SMD) to assess the covariable balance and reduce confounding variables. The co-variables were considered adequately balanced if SMD was calculated as<0.15, or else it would be adjusted for in the analysis. R 3.5.2 (R Foundation for Statistical Computing, Vienna, Austria) was used to conduct the propensity score matching.

### Statistical analysis

R version 3.5.2 (R Foundation for Statistical Computing, Vienna, Austria; www.R-project.org) was used to analyze the data. Two-tailed values of *P*<0.05 were considered statistically significant. Categorical variables are expressed as frequencies and percentages, and continuous variables are expressed as medians and interquartile ranges including the 25th and 75th percentiles or means and standard deviation. For comparative analysis between groups, the Mann-Whitney test or unpaired t-tests for continuous variables after testing for normality via the Shapiro-Wilk test and the Fisher’s exact test for categorical variables were used.

## Results

Overall, 113 children underwent the multistage rehabilitation and finally received complete repair between January 2016 and December 2018, in the 14 of which early extubation was performed. Of the originally identified 113 children, 3 patients were not able to be abstracted with all of the propensity score matching variables and were excluded from the study. Eventually, 14 patients receiving early extubation were successfully matched with 28 controls (Table 1). Propensity score matching successfully balanced the covariables in the matched subset (SMD < 0.2), which subsequently contributed to the comparable outcomes.

**Table 1:** Demographic and clinical variables pre- and post- propensity score matching

	Before matching			After matching		
	Early extubation (*n* = 14)	Unmatched controls (*n* = 96)	SMD	Early extubation (*n* = 14)	Matched controls (*n* = 28)	SMD
Age (y)	2.52 ± 2.07	3.55 ± 11.26	0.128	2.52 ± 2.07	2.68 ± 2.70	0.066
Weight (kg)	12.71 ± 4.05	12.64 ± 7.52	0.012	12.71 ± 4.05	13.74 ± 8.07	0.118
Male	3 (21.4%)	40 (41.7%)	0.452	3 (21.4%)	4 (14.3%)	0.187
MAPCAs	5 (35.7%)	48 (50%)	0.292	5 (35.7%)	12 (42.9%)	0.097
McGoon Index	1.75 ± 0.31	1.56 ± 0.38	0.534	1.75 ± 0.31	1.71 ± 0.45	0.105
Previous sternotomy	7 (50%)	40 (41.7%)	0.179	7 (50%)	13 (46.4%)	0.072
CPB time (min)	137 ± 39	160 ± 70	0.398	137 ± 39	137 ± 45	0.011
ACC time (min)	74 ± 28	87 ± 37	0.387	74 ± 28	73 ± 31	0.055
Perioperative VISmax	11.7 ± 4.1	13.0 ± 7.7	0.214	11.7 ± 4.1	11.5 ± 4.8	0.032
DSC	0 (0%)	2 (2.1%)	0.208	0 (0%)	0 (0%)	–
Fluid balance at 72 h postoperatively (mL)	− 336 ± 389	− 619 ± 780	0.460	−336 ± 389	− 343 ± 501	0.017
RV/LV pressure ratio	0.49 ± 0.09	0.50±0.14	0.011	0.49 ± 0.09	0.50±0.17	0.119

NOTE. Numeric variables: Mean ± SD

*Abbreviations*: *ACC* aorta cross clamping, *CPB* cardiopulmonary bypass, *MAPCAs* major aortopulmonary collaterals, *LV* left ventricular, *RV* right ventricular, *VISm* maximum vasoactive-inotropic score, *VSD* ventricular septal defect

Within the early extubation group, 5 (35.7%) of the patients were classified as type B PA/VSD/MAPCAs according to the classification of the Congenital Heart Surgery Nomenclature and Database Project, the rest of whom were classified into type A. Meanwhile, 7 (50%) of patients with early extubation received complete repair surgery after rehabilitation with others receiving one-stage repair surgery.

Compared with matched controls, patients in the early extubation group were demonstrated with a significantly reduced LOIS (1.9 [1.4, 3.8] d ν 4.1 [2.2, 10.9] d, *p* = 0.039) and pLOHS (9.0 [8.0, 15.3] d ν 17.0 [12.3, 28.0] d, *p* = 0.007). Meanwhile, the medical costs (69.5 [60.9, 144.8] × 1000CNY ν 17.0 [12.3, 28.0] × 1000CNY, *p* = 0.041) were significantly decreased in the early extubation group (Table 2).

**Table 2:** Outcomes associated with early extubation and postoperative complications

	Early extubation (*n* = 14)	Matched controls (*n* = 28)	*p* value
***Outcomes associated with UFTA***			
LOIS	1.9 (1.4, 3.8)	4.1 (2.2, 10.9)	0.039
Postoperative LOHS	9.0 (8.0, 15.3)	17.0 (12.3, 28.0)	0.007
Medical costs (× 1000CNY)	69.5 (60.9, 144.8)	113.6 (94.8, 183.1)	0.041
***Postoperative complications***			
Re-intubation	2 (14.3)	4 (14.3)	>0.999
Re-exploration	0 (0)	2 (7.1)	0.545
In-hospital mortality	0 (0)	1 (3.6)	>0.999
Lung complications	2 (14.3)	13 (46.4)	0.049
Arrhythmia	0 (0)	2 (7.1)	0.545
RRT	10 (71.4)	23 (88.5)	0.214

NOTE. Non-normally distributed variables: medians with 25th to 75th percentile

*Abbreviations*: *CNY* Chinese yuan, *LOHS* length of hospital stay, *LOIS* length of intensive care unit stay, *RRT* renal replacement therapy

As for the postoperative complications, no significant difference were detected between the two groups in re-intubation (14.3% ν 14.3%; OR 1.00; 95% CI 0.77–1.30; *p* > 0.999), re-exploration (0% ν 7.1%; OR 0.92; 95% CI 0.84–1.03; *p* = 0.545), in-hospital mortality (0% ν 3.6%; OR 0.96; 95% CI 0.90–1.04; *p* > 0.999), arrhythmia (0% ν 7.1%; OR 0.92; 95% CI 0.84–1.03; *p* = 0.545) and RRT (71.4% ν 88.5%; OR 0.63; 95% CI 0.20–1; *p* = 0.214) (Table 2). However, pulmonary complications (14.3% ν 46.4%; OR 0.63; 95% CI 0.42–0.94; *p* = 0.049) (Table 2) were with a significant lower rate in the early extubation group.

In addition, FFP (0 [0.0, 12.2] mL/kg ν 12.9 [0.0, 29.6] mL/kg, *p* = 0.041) transfusion volume were significantly reduced in the early extubation group with no significant difference was seen in PRBCs (15.5 [0.0, 32.8] mL/kg ν 22.2 [8.1, 54.2] mL/kg, *p* = 0.121) and platelets (0 [0.0, 6.8] mL/kg ν 22.2 [8.1, 54.2] mL/kg, *p* = 0.515) consumption (Table 3).

**Table 3:** Blood product consumption

	Early extubation (*n* = 14)	Matched controls (*n* = 28)	*p* value
PRBCs (mL/kg)	15.5 (0, 32.8)	22.2 (8.1, 54.2)	0.121
Platelets (mL/kg)	0 (0, 6.8)	0 (0, 7.6)	0.515
FFP (mL/kg)	0 (0, 12.2)	12.9 (0, 29.6)	0.041

NOTE. Non-normally distributed variables: medians with 25th to 75th percentile

*Abbreviations*: *FFP* fresh frozen plasma, *PRBCs* packed red blood cells

## Discussion

In this retrospective study, our data indicated early extubation may represent a primary strategy to improve early outcomes of patients after multistage rehabilitation and complex repair surgery of PA/VSD/MAPCAs. Early extubation is associated with earlier hospital discharge and shortening LOIS in such patients. Furthermore, positive economic effect on medical costs was either illustrated in patients with early extubation, which was accompanied by coincident and even much lower postoperative complications rates in the two groups.

Despite early extubation had been performed in varied congenital cardiac surgeries with specialized anesthesia management protocols and postoperative pain management [2, 8], the concept of early extubation for pediatric patients undergoing multistage rehabilitation and complete repair surgery of PA/VSD/MAPCAs has not been investigated individually in previous studies. The few studies about the postoperative short-term outcomes of patients with PA/VSD/MAPCAs focused on the predictors for prolonged mechanical ventilation time and whether it had influences on short-term outcomes such as in-hospital mortality or LOIS [9, 10]. The results of this study added some evidences to the limited researches, demonstrating both positive effects of early extubation on better short-term outcomes and economic benefits.

The safety and feasibility of early extubation in postcardiotomy pediatric patients for decreasing LOIS and reducing medical costs has been demonstrated in most of the simple congenital cardiac surgeries and even in the complex cardiac procedures such as the Fontan procedure and the Norwood procedure [11–13]. However, the re-intubation rate in the infants with early extubation was identified to be much higher in some researches [14, 15]. In fact, more than 95% of the patients elder than 1 year-old would remain a stable extubated condition after early extubation without increased postoperative complications [16]. Our cohort also illustrated the similar result that the 2 paients with re-intubation after early extubation were under 1 year-old, which resulted from reduced ventilation/diffusion function due to pulmonary infections. Referring to the rate of pulmonary infections, previous study has shown mechanical ventilation longer than 3 days in patients after cardiac surgery was associated with a significant increase in pulmonary infections [17]. Atelectasis, poor endotracheal suctioning and insufficient oral nursing care associated with mechanical ventilation through tracheal tube were wholly related to the development of lung complications, which made it non-controversial of the increased and worse lung complications in the patients with prolonged mechanical ventilation.

The negative effects of positive-pressure mechanical ventilation on patients were not restricted to pulmonary complications, which was either a leading cause to vasoactive agents consumption and fluid overload. In our study, we indeed observed a significantly decrease of FFP consumption in the early extubation group, which might resulted from shortened positive -pressure mechanical ventilation period. In addition, FFP consumption might directly induce the reopen of collateral vessels and the accumulation of colloid in the vessel bed, which is associated with the four postoperative occlusions in the matched control group.

Our study had some limitations that must be acknowledged. First, this study was a retrospective, observational study and involves a relatively small number of patients from a single center. Despite of the similar prevalence of postoperative complications other than lung complications, it is not enough to conclude the equivalent safety profile of early extubation compared with the matched control groups. Meanwhile, despite we all agree that postoperative outcomes of PA/VSD were closely associated with genetic anomalies, which occurred in 40% of such patients, the genetic testing was not carried out routinely in our institution. Therefore, we could not include it in the propensity score matching analysis. Second, considering the degree of difficulty of anesthetic management in the early extubation group, the anesthetist team and the surgeon team might be more experienced compared with the matched group. As a result, it might influence the outcomes of patients which would be not replicated in other researches. Third, the algorithm on selection of whether the patient was a candidate for early extubtaion was as follow. A comprehensive assessment of severity of cardiac defects, complexity and injury from surgical procedure, and pre- or intra-operative morbidities was made by the surgeon and the anesthesiologist together, especially the MAPCAs and its targeted therapy. If the surgical and perioperative team all agreed that the surgery was well-done and the MAPCAs were effectively managed, the early extubtaion was carried out immediately after surgery in the OR with the extubation protocol. However, the patients not acquiring a satisfied spontaneous breathing within 30 min after sternal closure were transferred to the ICU and eventually extubated within 6 h postoperatively. As mentioned above, early extubation were decided mainly based on the judgement of surgeons and anesthetists, which might result in a select bias towards a more stable patient in the early extubation group.

## Conclusions

Early extubation significantly reduced the LOIS and pLOHS in patients undergoing multistage pulmonary artery rehabilitation of PA/VSD without increasing any postoperative complications. Furthermore, less medical expenses associated with early extubation may relief the economic burden both for medical care program and patients’ family. Early extubation could be both feasible and valuable for patients with PA/VSD. It deserves further investigation to confirm the benefit of early extubation for such patiens in the multi-institutional controlled trials.

## Data Availability

All data generated or analysed during this study are included in this published article.

## Abbreviations

ACC: Aorta cross-clamping
CNY: Chinese Yuan
CPB: Cardiopulmonary bypass
FFP: Fresh frozen plasma
ICU: Intensive care unit
LOIS: Length of intensive care unit stay
MAPCAs: Major aorta-pulmonary collateral vessels
PA: Pulmonary atresia
PAs: Pulmonary arteries
PLOIS: Postoperative length of intensive care unit stay
PRBCs: Packed red blood cells
RRT: Renal replacement therapy
RV-PA: Right ventricle-pulmonary artery
SMD: Standard mean difference
VIS: Vasoactive inotropic score
VSD: Ventricular septal defect
